# Digital health literacy and subjective wellbeing in the context of COVID-19: A cross-sectional study among university students in Ecuador

**DOI:** 10.3389/fpubh.2022.1052423

**Published:** 2023-01-11

**Authors:** María F. Rivadeneira, Carmen Salvador, Lorena Araujo, José D. Caicedo-Gallardo, José Cóndor, Ana Lucía Torres-Castillo, María J. Miranda-Velasco, Kevin Dadaczynski, Orkan Okan

**Affiliations:** ^1^Faculty of Medicine, Public Health Institute, Pontificia Universidad Católica del Ecuador, Quito, Ecuador; ^2^Faculty of Medicine, Universidad Central del Ecuador, Quito, Ecuador; ^3^General Students Office, Pontificia Universidad Católica del Ecuador, Quito, Ecuador; ^4^Department of Education Sciences, Faculty of Teacher Training, University of Extremadura, Badajoz, Spain; ^5^Department of Health Science, Fulda University of Applied Sciences, Fulda, Germany; ^6^Centre for Applied Health Science, Leuphana University Lueneburg, Lüneburg, Germany; ^7^Department of Sport and Health Sciences, Technical University Munich, Munich, Germany

**Keywords:** digital health literacy, COVID-19, wellbeing, university, cross-sectional

## Abstract

**Background:**

The COVID-19 pandemic has generated an avalanche of information, which, if not properly addressed, generates uncertainty and limits healthy decision-making. On the other hand, the pandemic has exacerbated mental health problems among young people and adolescents, causing a worsening of their wellbeing. Previous studies have found that digital health literacy has a positive impact on people's attitudes toward the disease. This study aimed to analyze the association between digital health literacy on COVID-19 with subjective wellbeing in university students.

**Methods:**

A cross-sectional study was developed in 917 students from Ecuador. Subjective wellbeing was measured with the World Health Organization WellBeing Scale. Digital health literacy was assessed using the Spanish-translated version of the Digital Health Literacy Instrument adapted to the context of the COVID-19 pandemic. Bivariate and multivariate linear regressions were performed.

**Results:**

Digital health literacy and subjective wellbeing proofed to be significantly higher among males and among students with higher social status. The association between digital health literacy and subjective wellbeing was significant; for each increase of one point in the digital health literacy scale, an average increase of 9.64 points could be observed on the subjective wellbeing scale (IC 95% 5.61 – 13.67, *p*-value <0.001). This correlation persisted after adjust by demographic and socioeconomic variables.

**Conclusion:**

Improving digital health literacy in health would improve the subjective wellbeing of university students. It is suggested strengthen the digital health literacy through public and university policies that promote access, search skills and discernment of digital information. Socioeconomic and gender inequalities related to digital health literacy need to be further investigated.

## Introduction

The current COVID-19 pandemic has become a global societal challenge, causing an unprecedented public health crisis that in turn has led to a real economic catastrophe for many countries. COVID-19 is a new disease, and new knowledge is constantly being gained about its etiology, treatment, and prevention. Therefore, there is a high need for evidence-based, reliable, and trustworthy health information and sources.

The presence of the internet and its ability to massively and instantly spread information becomes an invaluable help in the fight against COVID-19, but it has also proved to be an obstacle by facilitating the fast spread of misinformation, falsehoods and myths (1). The World Health Organization has warned that we are not only facing a virus pandemic, but also a disinformation pandemic, and that both are equally dangerous (2). This “infodemic” or information epidemic, including both valid and false, is compounded by the fact that the authorized sources themselves were forced to change their recommendations as new information became known about this virus, its transmissibility, symptoms, and treatment (1).

Correct health information usually is communicated *via* health professionals and authorized publications. However, during the COVID-19 crisis a large amount of information on social networks and online media has been available that can overwhelm lay persons and professionals alike (3), who must have the ability to find relevant information, reflect critically, discern the information available and transfer it to everyday life. Research revealed during the initial phase of COVID-19, low-quality reviews were published at an accelerated rate, received considerable attention on academic and public platforms, which increased misinformation about the pandemic (4).

Appropriate use of information technologies is a valuable weapon to reduce the impact that this pandemic has on mental and physical health and the social wellbeing of individuals (5).

Appropriate use of information applies especially to university students, who make up a significant proportion of young adults worldwide and are constant users of the internet and social network sites. Health behavior in this period of life depends more on individual decisions and the life circumstances of students than on the guidance and help of parents or adults. Health literacy and digital health literacy is a central resource in this context (5–7).

Digital health literacy (DHL) is defined as “a set of fundamental skills that underlie the use of health-oriented information and communication technologies” (5). This allows individuals to search and understand information from electronic sources and apply this knowledge to promote, maintain or restore their own health (6). DHL is considered a determinant for health that not only favors the acquisition of knowledge but also promotes the active participation of citizens in health issues. Some skills inherent to digital health literature include operational and information use skills, but also strategic and communication skills (7).

DHL has a positive impact on people's attitudes toward disease and its prevention, thus improving their physical, mental and social health (7). A longitudinal study found that people with inadequate health literature had 230% increased odds over time for impaired psychological wellbeing (OR: 3.3, 95% CI: 1.70–6, 32, *p* < 0.001), adducing a positive longitudinal association between digital literature and health (8). A positive correlation has been found between higher digital health literacy score and better health, more healthy behaviors including disease prevention and management, etc. (9, 10). For example, willingness to be vaccinated against COVID-19 and the perception that infection could change personal lives, which in turn leads to better protective measures (8), is greater among those with better digital health literacy score. A better mental health has been found among those with better digital health literacy in health score (10, 11).

University students are considered a particularly vulnerable population with regard to mental health (12), with higher rates of depression and stress that affect their learning and quality of life (13). The pandemic has further exacerbated mental health problems among young people and adolescents, with a substantial increase in anxiety, depression, and distress, among other emotional problems (14, 15). Previous studies conducted by the COVID-HL Consortium (www.covid-hl.eu) (16) on digital health literacy in the context of the pandemic found that university students had low to very low levels of psychological wellbeing (12, 17, 18). Researchers suggest that psychological well-being is associated with DHL. The study carried out on university students in Vietnam shows a mediating role of DHL in the search for important information and psychological wellbeing, showing that a good DHL enhances the search for information for psychological wellbeing (18). For their part, Amoah et al. (19) found a positive association between DHL and psychological wellbeing during the COVID-19 pandemic in students from Hong Kong and Macao, although they warn that this association is not linear, but that socioeconomic status must be considered. Previous research also suggests differences in DHL by sociodemographic characteristics such as gender, age, etc. (18–20). However, information on DHL in Latin American students is not available so far.

The objective of this study was to assess the digital health literacy stratified by sociodemographic characteristics and to analyze its association with subjective wellbeing in Ecuadorian university students in the context of the COVID-19 pandemic. This study was carried out as part of the COVID-HL Consortium, a health literacy research network made up of 65 countries, with an emphasis on on different target groups (university students, school staff, COVID-survivors) and settings (university, school, health care) (https://covid-hl.eu) (16).

## Materials and methods

### Study design

A cross-sectional study performed in the confinement period due to COVID-19, with data collected from June 4 through 29 of 2020.

### Study setting and context

This study was conducted in Ecuador, a country where the first confirmed case of COVID-19 was recorded on February 29, 2020. It was the third country in Latin America to confirm COVID-19 cases, after Brazil and Mexico. COVID-19 cases spread rapidly throughout the country's provinces, making it one of the countries in Latin America hit hardest by the pandemic (21).

During the data collection period of the COVID-HL university student survey, from June 4 to 29, 2020, cases of COVID-19 had spread throughout the Ecuadorian territory, reaching 53,424 confirmed cases, with 8,026 deceased (22). Regarding the information policy, Ecuador has maintained control of official and epidemiological information by the current authorities throughout the pandemic. However, no policies have been established to monitor, regulate or measure the impact of the information circulated on social network sites. During the study period, onsite teaching in schools, high schools and universities was suspended by order of the supervisory authorities. All university courses were held remotely as online teaching.

The present study was carried out in two universities in Quito, the capital of Ecuador: the Central University of Ecuador (UCE) and the Pontifical Catholic University of Ecuador (PUCE). The public university (UCE) receives students from all over the national territory and from all socioeconomic classes. The private university (PUCE) receives students mostly from medium and medium-high socioeconomic classes.

### Sample and recruitment

Undergraduate, graduate and postgraduate university students from the UCE and the Quito headquarters of PUCE were invited to participate in an online survey. The online survey was applied through the platform Enterprise Feedback Suite survey tool by Tivian, software Unipark (https://www.unipark.com/). This software allows 10,000 respondents per project from each country, with high standards for data protection and security. All data are transmitted via an encrypted connection using the secure encryption protocol.

The study targeted all students enrolled in the June-December 2020 semester (*N* = 17,000). The sample was calculated from the following information: a size of the known universe of 17,000 students, an expected percentage of sufficient digital literature in university students of 49%, based on a previous study (20), with a level of 95% confidence and a sampling error of 5%. The minimum sample required based on this calculation was 601 students.

The recruitment process was as follows. An invitation was sent through the university institutional e-mail and the official and unofficial social networks of the aforementioned universities. Of the total number of students invited, 1,061 students agreed to participate. Those who gave consent to participate proceeded to read and approve the informed consent form, and then completed the survey online. The total number of students who completed the survey with valid responses was 917.

The information of the responses obtained online was stored anonymously, all data were exported and stored locally on a secure data infrastructure.

### Variables and measures

The dependent variable studied was subjective wellbeing during the last 2 weeks, which was measured with the World Health Organization (WHO) Wellbeing Scale (23, 24). This instrument includes five items that could be answered on a six-point Likert scale (from 0 = at no time to 5 = all the time). The value of each item is multiplied by four with a sum score ranging from 0 to 100, where 100 corresponds to the highest possible wellbeing (23, 24). The internal consistency of the subjective wellbeing scale was excellent (α =0.90, one-sided 95% CI 0.89). The internal consistency of the subjective wellbeing scale was high (Cronbach's alpha of 0.90, one-sided 95% CI 0.89).

The independent variable was Digital Health Literacy with respect to COVID-19 (DHLI). It was measured with the Spanish-translated version of the Digital Health Literacy Instrument (COVID-DHLI-Spanish) used by the Global COVID-HL Consortium (25), and based on the original version from van der Vaart and Drossaert (26). Dadaczynski et al. (16) adapted the instrument to the context of COVID-19 and used 5 of seven dimensions from the original DHLI scale (COVID-19 information search, adding self-generated content on COVID-19, evaluating reliability of COVID-19-related information, determining personal relevance of COVID-19-related information, and privacy protection on the internet). Each dimension includes three items to be answered on a four-point scale (1 = very difficult, 4 = very easy). The Spanish version was previously validated showing a good reliability (Cronbach's alpha 0.69, 95% CI 0.67) (27).

In accordance with previous literature that shows an association between wellbeing and socioeconomic and demographic variables (12, 13, 18, 19), the following were considered as adjustment covariates: gender (male/female), age (≤22 years/≥23 years), university (public/private), area of study of their major or degree, level of studies (undergraduate, master's degree, doctorate, etc.), presence of any chronic condition (yes/no) and subjective social status. To assess the subjective social status, the MacArthur scale developed by Adler et al. (28) and validated for the Spanish-speaking population (29) was applied. In this scale the illustration of a ladder is presented with 10 steps; respondents were asked to position themselves at the step that best reflected their status in the social hierarchy, with higher values indicating a higher social status.

In order to collect information on the variables described above, the online survey administered to university students contained the following: socioeconomic and demographic characteristics, the WHO wellbeing scale, and the Digital Health Literacy Instrument questionnaire adapted to Spanish (COVID- DHLI-Spanish). The entire survey was administered in Spanish, it was verified that the survey has adequate internal and construct validity (27). To reduce memory bias, related to self-reported studies, questions referring to the situation in the last 2 weeks were used, as well as re-questions to corroborate the information provided. In this research, data from students who answered the complete survey were included; no imputations of missing data were made.

### Statistical analysis

Kruskal-Wallis non-parametric mean difference tests were performed to analyze differences in COVID-19 related DHLI and subjective wellbeing scores between each of the covariates. The association between DHLI with each of the covariates was analyzed by applying bivariate linear regressions. The same analysis was applied for the subjective wellbeing scale. Those associations that maintained a value of *p* < 0.25 were entered in the multivariate analysis (30). Finally, a multivariate analysis was performed to confirm the association between DHLI with subjective wellbeing, adjusted for the variables that remained significant with a *p* < 0.05. Data were analyzed with Stata^®^ statistical software, version 15.0.

### Human participants and institutional review board

This research was approved by the Ethics Committee for Research in Human Beings of the Pontifical Catholic University of Ecuador, code EO-16-2020.

The participants were informed of the research and its objectives by e-mail, prior to signing the informed consent.

## Results

### Characteristics of the participants

Table 1 shows the socio-demographic characteristics of the sample. The 60.7% of respondents were female, the majority of them 22 years old or younger (67.28%), belonged to the public university (66.7%). Moreover, majority of the students were from the area of life and health sciences (85.71%), mainly undergraduate (94.66%) and did not have any chronic conditions (81.9%). 70.11% of them rated their social status between step 5 and 7 of the ladder (mean 5.2 SD ± 1.5).

**Table 1:** Characteristics of the sample.

**Characteristic**	***n* (%)**
**Sex**	
Female	557 (60.74)
Male	358 (39.04)
Diverse	2 (0.22)
**Age**	
≤ 22 years	617 (67.28)
≥23 years	300 (32.72)
**Type of university**	
Private	305 (33.30)
Public	611 (66.70)
**Study area**	
Engineering sciences and technologies	46 (5.02)
Life sciences and health	786 (85.71)
Basic sciences	19 (2.07)
Social sciences and humanities	65 (7.09)
Other	1 (0.11)
**Level of studies**	
Undergraduate	868 (94.66)
Master's degree	24 (2.62)
Other (a.e. Doctorate)	25 (2.73)
**Subjective social status**	
1–4	148 (16.14)
5–7	643 (70.12)
8–10	126 (13.74)
**Chronic condition**	
No	751 (81.9)
Yes	166 (18.1)

University students of Quito, Ecuador, 2020 (n = 917).

### Digital health literacy

**Figure 1:**
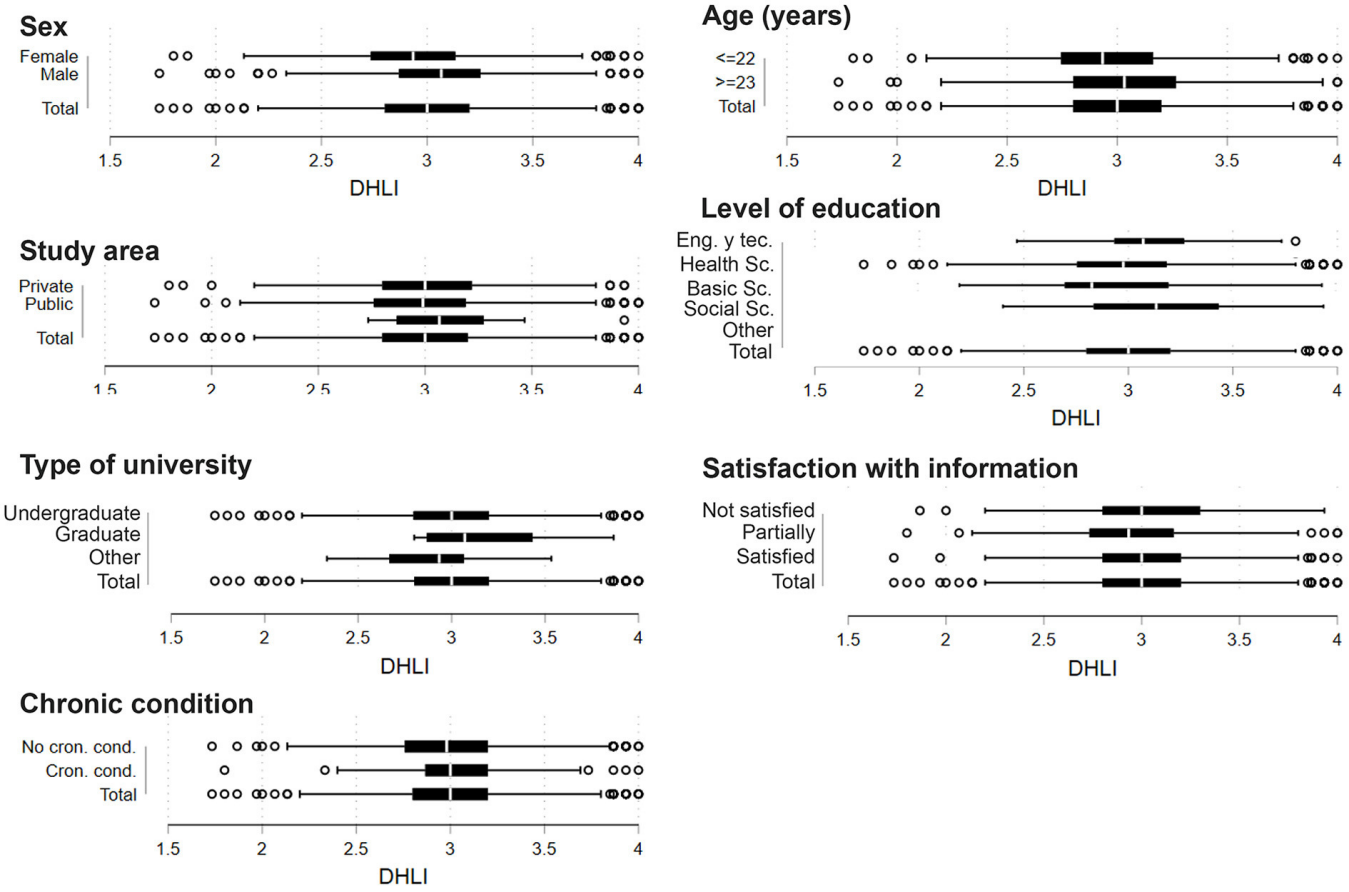
Box plot between DHLI scale and characteristics of university students. Ecuador, 2020 (*n* = 975).

On a scale from 1 to 4, the mean value digital health literacy across all dimensions was 2.9 (SD ± 0.5, Table 2). Figure 1 shows the box plots for the total COVID-19-related digital health literacy stratified by characteristics of the university students. Male university students had a significantly higher COVID-19-related digital health literacy than female students (3.0 SD ± 0.4 and 2.9 SD ± 0.3, respectively, *p* < 0.001). There were also significant age differences in the mean digital health literacy scores with older students showing a higher digital health literacy (3.0 SD ± 0.4 for students 23 years or older) as opposed to those of younger age (2.9 SD ± 0.3, *p* < 0.01). Moreover, those students from life and health sciences and basic sciences had a significantly lower digital health literacy compared to students from technological sciences and engineering (2.9 SD ± 0.4, 2.9 SD ± 0.4, and 3.1 SD ± 0.3, respectively, *p* < 0.05).

**Table 2:** Mean scores and Cronbach's alpha for the DHL and subjective wellbeing scales.

	**Mean (SD)**	**Alfa de Cronbach**	**95% CI Unilateral**
Digital health literacy	2.91 (0.53)	0.742	0.711
Global	2.81 (0.54)	0.777	0.754
Information search	2.9 (0.55)	0.721	0.691
Auto-generated	2.95 (0.5)	0.741	0.713
content			
Reliability	3.37 (0.51)	0.728	0.699
Relevance	2.99 (0.36)	0.457	0.399
Privacy protection	3.63 (0.71)	0.903	0.895
Subjective wellbeing scale	53.2 (20.7)	0.697	0.668

Further bivariate linear regression analyses showed that male students and those aged 23 years or older had a significantly higher digital health literacy (Beta coefficient = 0.1, 95% CI 0.08–0.2, *p* < 0.01; 0.07 95% CI 0.02–0.1, *p* < 0.01). A higher subjective social status was positively associated with a higher COVID-19-related digital health literacy. For each point of increase in subjective social status, there was an average increase of 0.05 on the digital health literacy scale (Beta coefficient = 0.05, 95% CI 0.03–0.06, *p* < 0.001) (Table 3). After adjustment, the variables gender (with a higher DHL score for men compared to women) and social status (with a higher LHD score for higher perceived social status), remained significant with a *p* < 0.05.

**Table 3:** Digital health literacy and characteristics of university students.

	**Beta coefficient**	**95% CI**	***p*-value**
**Sex**			
Female	Reference		
Male	0.13	0.08–0.18	< 0.001^**^
**Age**			
≤ 22 years	Reference		
≥23 years	0.07	0.02–0.13	0.008^**^
**Type of university**			
Private	Reference		
Public	−0.04	−0.10–0.01	0.144
**Study area**			
Engineering sciences and technologies	Reference		
Life sciences and health	−0.12	−0.01–0.25	0.073
Basic sciences	−0.06	−0.22–0.11	0.469
Social sciences and humanities	−0.14	−0.04–0.25	0.006^**^
Other	−0.1	−0.8–0.59	0.772
**Level of studies**			
Undergraduate	Reference		
Master's degree	0.16	−0.01–0.34	0.067
Other (a.e. Doctorate)	−0.09	−0.24–0.05	0.198
**Semester in undergraduate**			
≤ 2nd semester	Reference		
≥3rd semester	−0.02	−0.11–0.06	0.572
**Subjective social status (10 steps)**			
Score (1–10)	0.05	0.03–0.06	< 0.001^**^
**Chronic condition**			
No	Reference		
Yes	0.05	−0.01–0.12	0.124

** Significant p < 0.01.

Bivariate linear regression. University students from Quito, Ecuador, 2020.

### Subjective wellbeing and associated variables

**Figure 2:**
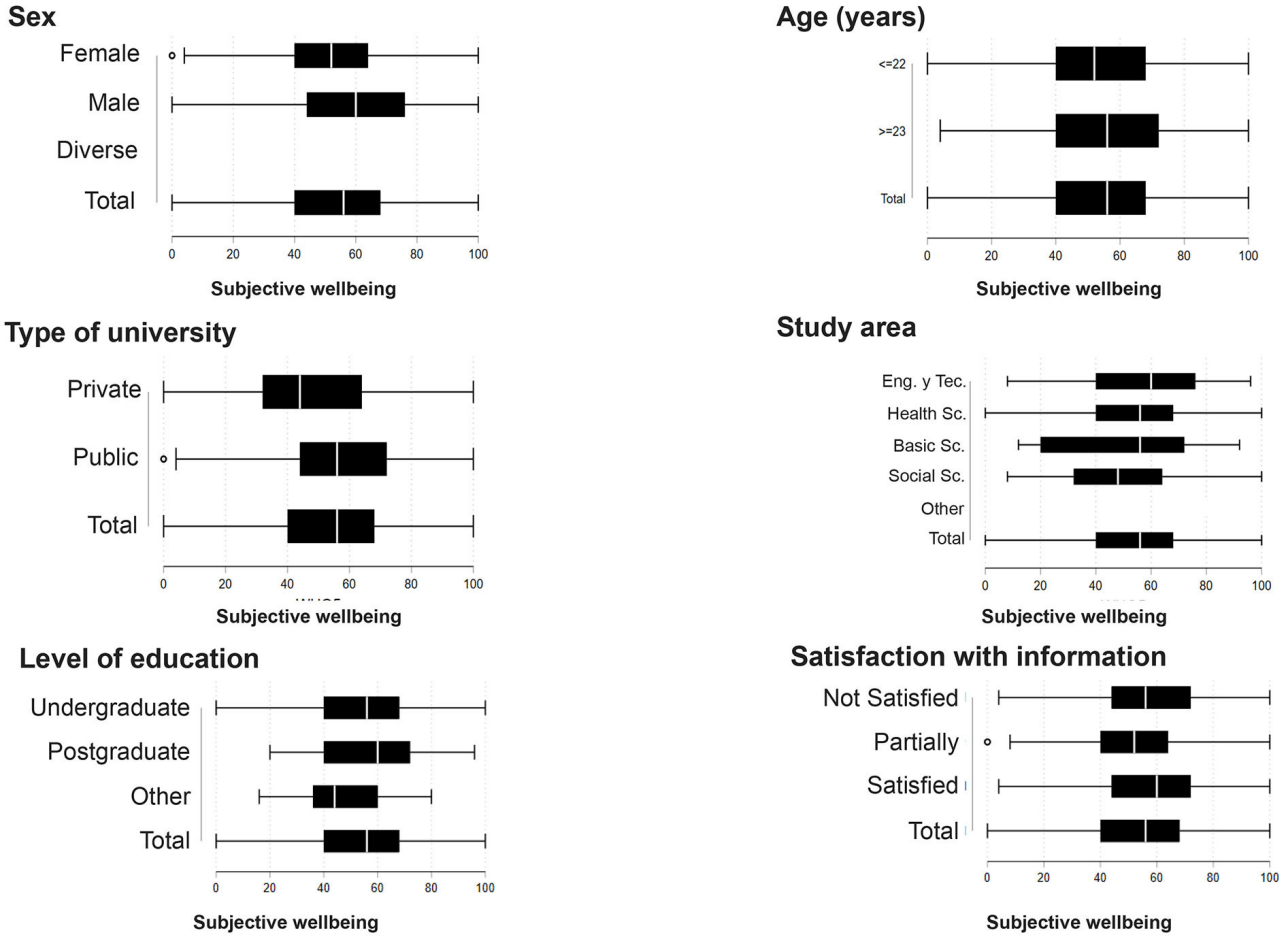
Box plots between the subjective wellbeing scale and characteristics of the university students. Ecuador, 2020.

On a 0 to 100 scale, subjective well-being reached a mean of 53.2 SD ± 20.7 (Table 2). Males reported a significant higher subjective well-being than females (58.4 SD ± 20.5, and 50 SD ± 19.9, respectively, *p* < 0.001) (Figure 2). Students 23 years of age or older had a significantly higher subjective wellbeing compared to younger students (55.5 SD ± 20.2, and 52 SD ± 20.7, respectively, *p* 0.01). Moreover, the wellbeing mean value was significantly higher for students in the public university enrolled at a private university (55.8 SD ± 19.2, and 47.9 SD ± 22.4, respectively, *p* < 0.001). Similarly, there are significant lower wellbeing means for social science students as compared to those of technological sciences and engineering (50.2 SD ± 21.8, and 57.8 SD ± 23.1, *p* < 0.05). Students with chronic diseases also reported a lower subjective wellbeing score than those respondents without a chronic condition (49.6 SD + 21.9, and 53.9 + 20.3, respectively, *p* < 0.05).

In further regression analyses it was found that, on average, males have 5.26 higher point on the subjective well-being scale (95% CI 2.52 − 7.99, *p*<0.001 than females. Moreover, compared to respondents from private universities, public university students reported a 11.12 points higher subjective well-being (95% CI 7.54 − 14.71, *p* < 0.001). Life and health sciences and Basic sciences students had a subjective well-being score that was 15.39 points lower (95% CI −29.68 − −1.11, *p*-value 0.03). These associations remained significant after multivariate adjustment (Table 4).

**Table 4:** Correlations between subjective wellbeing, DHL and characteristics of the participants.

	**Bivariate linear regression**			**Multiple linear regression**		
	**Coefficient**	**95% CI**	***p*** **-value**	**Coefficient**	**95% CI**	***p*** **-value**
**Sex**						
Female	Ref.			Ref.		
Male	8.41	5.71 – 11.11	< 0.01^**^	5.26	2.52–7.99	< 0.01^**^
**Age**						
≤ 22 years	Ref.			Ref.		
≥ 23 years	3.52	0.66–6.37	0.02	2.57	0.35–5.51	0.09
**Type of university**						
Private	Ref.			Ref.		
Public	7.87	4.91–10.82	< 0.01^**^	11.12	7.54–14.71	< 0.001^***^
**Study area**						
Engineering sciences and technologies	Ref.			Ref.		
Life sciences and health	4.73	−2.33–11.39	0.19	−7.85	−15.87–16.00	0.05
Basic sciences	−5.34	−17.84–7.34	0.41	−15.39	−29.68–−1.11	0.03^*^
Social sciences and humanities	−3.02	−8.58–2.52	0.29	−3.88	−13.19–5.44	0.41
Other	26.75	25.33–28.16	< 0.001^***^	21.26	13.25–29.27	< 0.001^***^
**Level of studies**						
Undergraduate	Ref.					
Master's degree	4.81	−3.58–13.19	0.26			
Other (a.e. Doctorate)	−4.87	−13.09–3.35	0.25			
**Subjective social status (10 steps)**						
Score (1–10)	3.71	2.79–4.62	< 0.001^**^	2.83	1.88–3.77	< 0.001^**^
**Chronic condition**						
No	Ref.			Ref.		
Yes	−4.34	−7.81– −0.86	< 0.01^**^	−2.73	−6.43–0.96	0.15^*^
**DHLI scale**						
DHLI score	13.18	9.25 - 17.10	< 0.001^***^	9.64	5.61–13.67	< 0.001^**^

* Significant p < 0.05,

** significant p < 0.01,

*** significant p < 0.001.

With regard to subjective social status, a positive and significant relationship with subjective wellbeing was found. For every one-point increase in subjective social status, the subjective wellbeing scale increased by an average of 2.83 points. (95% CI 1.88 – 3.77, *p* < 0.001). Having a chronic health condition decreased the subjective wellbeing score by an average of 4.34 points (95% CI −7.81 − −0.86, *p* < 0.05). The relationship with subjective wellbeing remained significant in the multiple linear regression (Table 4).

### Subjective wellbeing and DHLI

The association between DHLI and subjective wellbeing was significant; per each one-point increase on the DHLI scale, there is an average increase of 9.64 points on the subjective well-being scale (95% CI 5.61 − 13.67, *p* <0.001). This association remained significant after adjusting for the covariates, i.e., the correlation between digital health literacy and subjective wellbeing is independent of gender, age, university, study area, subjective social status, and the presence or absence of chronic illness (Table 4).

## Discussion

Digital health literacy comprises a set of skills for searching, understanding, communicating, critically evaluating and applying the information retrieved from digital media. Linking digital health literacy with mental provides a basis to better understand the determinants of health especially in times of crisis such as the COVID-19 pandemic (31).

Since 2010, Ecuador has sought to promote the digital literacy of its inhabitants (32). Efforts were made to equip educational institutions with computers and incorporate New Communication and Information Technologies (NICTs, as the Government called them) to the teaching-learning process, including teacher-training processes to manage them. The indicators are still very low in the formal education sector. As of 2010, 32.4% of rural educational institutions and 38.7% of urban institutions at different educational levels (basic, high school, university, etc.) had free internet service. Even so, it is already a noticeable improvement compared to 2008, when only 10% of rural institutions offered internet. A notable part of this increase was the construction in the rural sector of institutions called “Millennium Educational Units,” specifically conceived to take advantage of the NICTs (33).

However, the aforementioned advances were not specifically focused on health issues. Therefore, it is important to emphasize the importance of adequate digital health literacy, especially during this COVID-19 pandemic, both on a personal and a community level, given the increase in false information and news that negatively influence the prevention of diseases and the promotion of health (34).

Along with this, adequate digital health literacy is essential for the care of mental health and wellbeing of people, because the positive behavioral changes that information in digital media generates are remarkable, fast, and practical (35). For example, the information on the use of masks as something daily, the use of alcohol and disinfectant gel, social distancing, and staying at home, reinforced the prevention of contagion (36).

In this study, on a scale from 1 to 4, the mean value digital health literacy literacy across all dimensions 2.9 (SD ± 0.5). In Germany, levels of digital health literacy in health reported during 2020 reached a 49.9% for sufficient DHL, 34.9% for problematic, and 15.2%for inadequate DHL (31), and it also coincides with what was found in the United States and Pakistan, where 49 and 54.3%, respectively, of university students have adequate digital health literacy and health literacy (10, 37).

This study showed significantly higher levels of digital health literacy in male students than in female respondents, which coincides with previous studies in Palestine (38) and Portugal (39), although the inverse relationship has also been identified in other studies (40–42). Previous studies have suggested that female students would face greater difficulties in analyzing and trusting the available information, when compared to male, which could affect their level of digital health literacy (20, 39). As mentioned by other authors (20, 39), women are the ones who often bear the greatest responsibilities in relation to family care, particularly health care, for which reason they seek various sources of information in this regard. This could lead to them being more critical of information about COVID-19, and more sensitive about the reliability of the information available.

Better digital health literacy was found among university students older than 23 years, which is consistent with other studies (31, 42); however, some studies mention opposite results (43). Probably, the higher level of DHL in older students found in the present study has to do with an increase in their abilities to discern information relevant to their health as their university education also progresses, which would imply a positive role of the educational level at DHL (34, 41).

On the other hand, higher scores in digital health literacy were found in students who reported better subjective social status, which can be considered a proxy for socioeconomic status, which is consistent with previous findings (11, 12). This finding suggests the importance of taking into account the socio-economic background as a possible factor that ends the level of DHL. Lower-income students are likely to have less access and less ability to search for, understand, and disseminate relevant information.

Regarding subjective wellbeing, higher scores were found in male students. Other studies carried out during the period of the COVID-19 pandemic reveal a greater deterioration in psychological wellbeing in women, which is observed along with a greater burden of domestic work, greater job losses, and a reduction in economic income (44–46). Particularly, in a study carried out on university students, women presented higher rates of loneliness, stress and anxiety than men (47). Higher psychological wellbeing scores were found in students with better socioeconomic status, which is similar to other studies (47, 48). Fogel et al. (49) and Amoah et al. (19) obtained similar data to this study concerning the association between digital health literacy and income.

The population in this study is university students, which has certain advantages over other population groups due to the level of education they have attained. Adil and colleagues have found that educational level is the main factor for the uneven response toward digital health literacy (50). In this sense, achieving better DHL levels requires improving the education levels of the general population.

A positive relationship was found between the digital health literacy related to COVID-19 and subjective wellbeing. Previous studies have found similar results. For example, having better digital health literacy in times of COVID-19 was negatively associated with the probability of having depression (OR 0.9, *p* < 0.001), and positively with Health-Related Quality of Life (β = 0.5, *P* < 0.001; β = 0.8, *p* < 0.001) in a (include country) study with (include population) (51, 52). On contrary a study from Singapore found the opposite i.e., individuals with higher digital health literacy had a greater decline in their subjective wellbeing (ordinal probit, β = −0.02, *p* < 0.05 for February 2020, and β = −0.08, *p* < 0.001 for July 2020). However, the authors emphasize that this finding may be due to socioeconomic status (53). Other research on medical students in Vietnam found an association between lower health literacy during the pandemic and greater fear of COVID-19 (β = −0.06, *p* < 0.001) (18).

These results would imply that people with higher digital health literacy use certain digital sources, such as social media, less often and are better able to find and use high-quality health information that is beneficial in protecting them from infection with the coronavirus and help them to promote your wellbeing.

An interesting observation is that although the pandemic has been managed differently all around the world and COVID-19 prevalence is very different between countries; the experience of college students may be comparable between the countries. Gender differences in COVID-19-related concerns have been demonstrated, with female college students scoring significantly higher than male students on depression, anxiety, and stress do during the early stages of the pandemic (36). This study also found that younger adult students (ages 18–24) had more symptoms of anxiety and depression during COVID-19 than older adult students (≥25 years old), which supports our findings on differences between undergraduate and graduate students.

Although the evidence shows gender and social status differences in stress and anxiety prior to the pandemic (38), the pandemic may have amplified these discrepancies. For example, competing demands for caregiving responsibilities and studying online are more likely to affect women than men, and people with lower social status may not have the same access to the resources they need or the adequate Internet connectivity to allow them to study online (12).

For this reason, it is considered important to improve digital health literacy of university students. Access to high quality information from reliable sources, and digital literacy that is adequate to distinguish between fake news and information with scientific evidence, are essential. It is important that university students and the general public are able to analyze, evaluate and apply the information they find in digital media, and consequently, protect their health and that of their family and community. Additionally, digital literacy may well be considered as a fundamental part of people's responsibility and social conscience (5, 6), both from those who create and spread the information and from those who receive it. University and government policies should aim to reduce socioeconomic and gender inequalities in terms of digital health literacy.

This research has several limitations: first, the survey was conducted three and a half months after the rapid spread of COVID-19 in Ecuador, which could have intensified fear and decreased the student's satisfaction with life. Another limitation has to do with the respondents and convenience sampling; most of the responses were from health and life sciences students, which could generate a bias in the results obtained, since they, in general terms, have more information on health-related topics. In this study, wellbeing was measured through a subjective assessment in the last 2 weeks, and other variables that could be related to wellbeing, such as grief, mental health illnesses, job layoffs, family support, etc., were not considered. The low response rate in this study may be due to the technical difficulties encountered at the beginning of the pandemic to gain better acceptance among students, such as access to technical resources and the use of online surveys, which they were not familiar with.

However, this research is part of a series of articles on digital health literacy in health related to COVID-19, in international cooperation between 65 countries sponsored by the Consortium of COVID-HL Universities (https://covid-hl.eu) (16). This will allow future comparison between countries. The questions in digital health literacy in health and their respective scale were previously validated in a European context, and later, in the Ecuadorian context. Likewise, the different methodologies used are simple, reliable and robust, which facilitate their replication and adjustment.

## Conclusion

Access to quality digital information and the skills to search for useful information, as well as their discernment for making health decisions, are key elements in protecting and promoting health (5, 9, 10). This is particularly important in the context of the COVID-19 pandemic, where a large amount of digital information has been available from the outset, resulting in information avalanche that could lead to misinformation and misguided health decisions (3, 4). In this sense, digital health literacy in health becomes important. Even more important is for adolescents and young adults, frequent users of digital social media, who, in turn, transmit this information to their peers, their families and communities; and who, at the same time, have been one of the groups most affected in their mental and social health due to the pandemic (12–15).

In this study, the subjective wellbeing perceived by university students in Ecuador was significantly associated with digital health literacy related to COVID-19. This finding could suggest that efforts to improve digital literature in university students could potentially be related to improving their well-being, and vice versa.

It is necessary to make visible the importance of digital health literacy for the promotion and prevention of health, particularly in the current pandemic. Coordinated public and private efforts are required to reinforce digital health literacy in health among adults and young adults, by recognizing the importance of skills to navigate the internet and other digital environments in order to promote both personal and community health. Special attention must be devoted to the university population, as well as socioeconomic and gender inequalities must be addressed. This study also suggests gender and socioeconomic inequalities related to digital health literacy, so further research on these gaps is required. This analysis will be part of a future investigation that the authors plan to carry out.

## Data availability statement

The datasets presented in this study can be found in online repositories. The names of the repository/repositories and accession number(s) can be found in the article/supplementary material.

## Ethics statement

The studies involving human participants were reviewed and approved by Ethics Committee for Research in Human Beings of the Pontifical Catholic University of Ecuador. The patients/participants provided their written informed consent to participate in this study.

## Author contributions

MR designed and executed the study, developed the data analysis model, and wrote the preliminary draft. CS helped with study design, coordinated data collection, and contributed to writing the preliminary draft. JC-G performed the validation of the scales and the statistical analysis and participated in the writing of the draft. LA, JC, AT-C, MM-V, KD, and OO developed the instrument and overall study design, coordinated data collection, and made important contributions to writing this scientific article. All authors contributed to the article and approved the submitted version.
